# Hospital and Institutional News

**Published:** 1912-11-30

**Authors:** 


### HOSPITAL AND INSTITUTIONAL NEWS.

### WHERE IS THE BRITISH HOSPITALS ASSOCIATION?

Mr. Walter Alvey's repetition of our own constant warning that the hospital world must lose not a single further moment in taking combined action to meet the grave situation which will arise on January 15, has led to the following anxious question, which is being asked on all sides: "Where is the British Hospitals Association?" Since the successful close of its conference at Birmingham two months ago the Association has made no sign of any kind. Why is it silent and inactive at this important crisis in the history of the voluntary hospitals? All over the country its inactivity is causing serious dismay. And yet no one needs reminding that when the Insurance Act was still a Bill the Association was sufficiently active. It held two great meetings in London. It sent a deputation to the Chancellor of the Exchequer: it drew a statement of the utmost importance from him. A mandate for leadership was thrown at it with both hands. At very short notice two hundred voluntary hospitals sent their representatives to its emergency meetings to consider what was to be done. In other words, at the first sign of danger the British Hospitals Association showed that it had a useful purpose to fulfil. Are we to be compelled to add that now, when the battle for the voluntary system is far more urgent, the Association is asleep, or moribund, or at any rate quite inactive? Has it not an Executive Committee? Has this been called together, and what is the British Hospitals Association going to do? That it should have allowed Mr. Alvey's complaint of non-co-operation and apathy to pass without an instant challenge from its secretary setting forth what its purpose and policy are is symptomatic. There could never be a finer opportunity. What, then, is the Association about?

### IS LISTER ALREADY FORGOTTEN?

The latest step in the progress of the Lister Memorial Scheme is the issue of a circular by the chairman, treasurers, and secretary of the Fund. After recalling that the three proposals for the form of the memorial were a medallion and inscription in the Abbey, a monument in a public place in London, and an international fund for the advancement of surgery, the writers state that it is proposed to form committees in the provinces, dependencies of the Empire, and in foreign countries to co-ordinate the collection of subscriptions. The treasurers of the Lister Memorial Fund, Royal Society, Burlington Gardens, are ready to receive subscriptions. Since it cannot be denied that even the Memorial Fund to Florence Nightingale was in serious danger until a whip was given, which The Hospital was the first to crack, we hope that the claims of the Lister Memorial will be enforced vigorously. For two criticisms almost always with justice can be directed at every memorial scheme. The first is that invaluable time is wasted before the memorial committee meets and issues its appeal, and the second is that those responsible for important memorial schemes hardly ever remember how short the public's memory really is. Among the workers of the hospital world the names of Florence Nightingale and Lord Lister have a unique place; among the general public they cannot be expected to command the same reverence and love; and yet it is the general mass of the public which is appealed to for subscriptions. Not a further moment should be lost. While the public is being reminded of the achievements of the great man or woman that has died the appeal should be issued. Every week's delay means a loss of many hundreds of pounds. If anyone doubts this let him ask himself if the *Titanic* Memorial Fund would stand any chance of reaching its present amount of some half-a-million if it had not been opened until to-day. We hope, therefore, that the Lister Memorial Committee will keep its claim in the eye of public opinion without ceasing until the necessary sums have been raised.

### A GLASGOW WARD AS A LISTER MUSEUM.

The suggestion has come from Glasgow that one of the wards of the Royal Infirmary there, where Lord Lister first experimented with antiseptic methods, should be set apart as a museum in which objects of interest associated with the man and his discoveries might be preserved on exhibition. The committee of the Lister Memorial Fund have been informed that the directors of the infirmary have sanctioned this scheme, and objects suitable for the project are therefore solicited, and will be gladly received by the Superintendent of the Royal Infirmary, Glasgow. This proposal, which is a very natural one, is of more than obvious interest, though impracticable as Glasgow requires more beds than the Infirmary contains. It must also be considered in the light of Lord Lister's will, which showed not merely extraordinary modesty, but even an anxiety to escape posthumous fame. For example, he did not wish his name "in any way to be associated" with his gifts to the King's Fund and certain hospitals. He gave his nephews power to destroy as many of his scientific papers as they deemed of no scientific value—that is, in effect, as many as they liked. It is true that he left his orders and medals to the University of Edinburgh, but this bequest was accompanied by the express proviso that it may dispose of all or any of them, "for example, by having the medals melted down or the diplomas or other writings destroyed," should it at any time wish to do so. Few testators have ever given so strong a hint that they are indifferent to the preservation of their gifts. It is, therefore, rather curious that some seven months after the publication of a will of this exceptionally modest kind a proposal should be made to preserve the very things to the fate of which the testator showed he was indifferent. Still, a public man is not merely the idol but the possession of the public, which exacts a very real control in return for the rewards and the praise which it bestows. The proposed museum is an instance of this control, which is worth recording.

### SEGREGATION AS A PANACEA.

Lord Dunedin opened on Saturday last the new dispensary of the Royal Victoria Hospital for Consumption in Lady Lawson Street, Edinburgh. The chief feature of his and the subsequent speech made by Sir Malcolm Morris was the tribute both paid to the work of Dr. Philip and his lay co-operators in Edinburgh. Sir Malcolm Morris, who is one of the founders of the National Association for the Prevention of Consumption, said that without Dr. Philip's work the Chancellor of the Exchequer would never have dared to touch tuberculosis, nor would the disease have been deemed suitable for inclusion in the Insurance Act. In the evening a demonstration of the Edinburgh methods for the prevention and treatment of consumption was given, at which Sir Malcolm Morris again spoke, urging that it would be far safer if persons in the advanced stages of consumption would allow themselves to be separated from their homes and sent to a home. It seems to us that this counsel of perfection depends very much on the sort of home from which the unfortunate patients come. In the case of the poor, whose conditions of life are often so unhealthy as to give plenty of excuse for the hygienically minded to demand the dispersion of the family, there may be much said in favour of this drastic proposal. But it is idle to deny that the better-off classes, with the means and expert advice that they have at their disposal, can and do take sufficient precautions to make Sir Malcolm's suggestion supererogatory. Segregation as a panacea is as dangerous as any other cry, and there seems a tendency abroad to prescribe it somewhat excessively.

### THE KING'S COLLEGE HOSPITAL BUILDINGS.

It seems that the Metropolitan Water Board may become the new owners of the King's College Hospital site and buildings in Portugal Street, Lincoln's Inn. At a recent meeting of the Board the General Purposes Committee proposed that they should be authorised to negotiate for the acquisition of the property for the purpose of converting the buildings into new central offices for the Board. They reported that the hospital stood on what was practically an island site with an area of about 48,000 square feet. The buildings are to be vacated in about a year's time, and the Committee considered that the existing premises could be adapted so as to provide ample accommodation for the present staff and for future requirements, if the property could be acquired at a reasonable cost. As yet the Committee are not in a position to state what sum would be accepted for the property, but they consider that the proposal would be much more economical than the provision of a new building. Mr. Fitzroy Doll, however, said that it was ridiculous to suggest that the hospital could be converted into appropriate office premises, and after some discussion consideration of the proposal was adjourned.

### LONDON'S MENTAL HOSPITAL PATIENTS.

In The Hospital of July 6 it was stated that the London County Council were about to enter into new contracts for the accommodation of London's mental hospital patients in other than London county mental hospitals. Since that date contracts have been entered into with Fisherton House, Salisbury, for the reception of twenty-five males; with Monmouthshire, Abergavenny, for twenty-five males and fifty females; with Leicester Borough Mental Hospital at West Humberstone for sixty females; and with Cornwall County, Bodmin, for twenty-five males. The period of the contract varies from three to five years, and the rate varies from 14s. to 15s. 2d. per week. It is also interesting to note that the retirement in May last of the steward of Cane Hill Mental Hospital has enabled the London County Council to use the house which he occupied on the estate as a villa, to be known as Postern Villa, for fourteen female patients under the charge of a resident nurse.

### THE EARLY HISTORY OF THE WESTMINSTER HOSPITAL.

The proposed removal of the Westminster Hospital, some comments upon which are offered in our leading article this week (p. 232), is not by any means the first in the history of the institution. Founded as the Westminster Infirmary in December 1719, the original premises were in Petty France: here the first out-patient was treated in February 1720, and the first in-patient was received in May of the same year. Then in 1724 the Westminster Public Infirmary, as it was by that time called, was removed to Chapel Street, and thence to James Street in 1734. In the previous year, when this move was in contemplation, occurred the historic split which resulted in the secession of the whole of the medical staff and several of the governors, and the foundation by them of St. George's Hospital in Lanesborough House at Hyde Park Corner. The present buildings at Westminster date from 1834, and extensions took place in 1886-7 and in 1900-1. Coronations and other pageants focussed in the Abbey have in the past been a fertile source of revenue to the institution in Broad Sanctuary through the erection of stands and the letting of seats.

### ADDENBROOKE'S HOSPITAL, CAMBRIDGE.

The most interesting business at the Quarterly Court of Governors of this hospital held the other day was that the plans of the new clinical laboratory which Mrs. Burnett is erecting, equipping, and endowing in memory of her late son, Mr. John Burnett, have been approved and that a tender for the work has been accepted. The sum of £3,000 will be expended on this laboratory, and an endowment of £350 a year is being provided by the generosity of the giver. At Cambridge the relations between hospital and school are even less formal and more nebulous than at most teaching hospitals. As a rule the necessary pathological and bacteriological work is done in the University laboratories, which are at some little distance from the hospital. The new arrangement will make the hospital more independent, and will at the same time benefit the University by providing adequate remuneration for officials who are as a rule none too well paid. Another interesting item on the agenda was the receipt of £477 from the Cambridge and District Workers Hospital Fund, an organisation whose commendable energy and success we have noted on a previous occasion; and of some £35 from the Cambridge Bijou Amateur Dramatic Club. Addenbrooke's is rich in friends, for yet another collecting organisation has handed in £391.

### THE WORCESTER MEMORIAL TO KING EDWARD.

Earlier this month the Bishop of Worcester formally opened the new annexe to the Worcester General Infirmary, which is the city's memorial to the late King. From the speeches delivered by the Bishop, the Mayor, and others at the opening ceremony, it appears that the medical staff petitioned for further accommodation three years ago. Then the matron and nurses collected £68 towards improvements in the children's wards, thus setting the ball rolling. A sub-committee then determined on the requirements, and when these had been reported to the governors it was decided to issue an appeal at once. At this stage it became known that local opinion favoured the fusion of the scheme with that for a memorial to King Edward VII. The gravest of the financial difficulties thus solved, the work was put in hand, and has cost in buildings and equipment nearly £2,500. Some £1,456 of this has been collected by the Memorial Committee, but £600 was still required on the opening day to liquidate the debt completely. The new wing is at the east end of the infirmary. It includes three consulting rooms with examining rooms adjoining, a dentist's room, laboratory, nurses' room, casualty room, *x*-ray room, and an out-patient operating room, all entered from a wide corridor which opens off the end of the waiting hall. The walls of this corridor and of the operating room have glazed tile dadoes, and all the floors are jointless and impervious. The annexe has a flat asphalte roof, enclosed by parapet and railings; on to it the first floor children's ward opens, and it is intended to utilise it for the open-air treatment of the children. To this end part of it has been covered in to form a shelter with a south aspect.

### BACTERIOLOGY IN RED CROSS WORK.

The British Red Cross Society has been well advised to enforce its claims for support by reminding the public that its work does not begin and end on the field of battle. The newspapers have been full of late of reports concerning the presence of cholera and typhus in Constantinople, reports which the Society has stated to be confirmed by its Red Cross Director for Turkey. On Tuesday last, therefore, it arranged that Captain J. H. Horton, D.S.O., of the Indian Medical Service, with Captain A. B. Smallman, R.A.M.C., as bacteriologist from the laboratory of the Royal Army Medical College, and Captain Lloyd Jones, R.A.M.C., should start for Constantinople. The party, it is stated, will be fully equipped with the means for carrying out bacteriological work, taking in fact some ten tons of food and stores with them. They will not arrive at Constantinople as an isolated unit, since two base hospitals have already been opened there, and the Society has at present in the city nine medical officers, nine dressers, and thirty-six trained orderlies. Epidemics cannot, however, be controlled in a moment, and though the close of the war will mean the first step towards restoring smooth working generally, the Society is likely to be kept busy for a considerable time after peace has been declared.

### A FREE TUBERCULIN DISPENSARY OPENED.

Professor Annett, of the Liverpool School of Tropical Medicine, has now opened at Runcorn, his native place, a tuberculosis dispensary, where, with the help of his assistants, he will give free treatment to poor patients by means of tuberculin. The dispensary, at which inoculations will be given twice a week, possesses accommodation for some thirty to forty patients. Ten were present at the opening of the dispensary. Additional gratitude will be felt for the Professor's action if to the freedom treatment that has been started is added a record of the results attained by the tuberculin method. Every new centre of information concerning this much debated treatment is of value, and we hope that the history of the dispensary patients will be carefully followed, recorded, and published. The evidence of experiment is still necessary, and doubtless will be forthcoming from the new centre at Runcorn.

### PHARMACISTS AND THE CONFERENCE.

The work connected with the British Pharmaceutical Conference of 1913 has already been commenced in earnest. The meeting is to be held in London in July; and, being the Jubilee gathering, will be of unusual interest. We are glad to note that institution pharmacists are taking their place in the work of organising and are well represented. The committee, of which Mr. Edmund White, B.Sc., F.I.C. ex-pharmacist, St. Thomas's Hospital, is chairman, includes Mr. R. W. Lindsey, F.C.S., St. Mary's, Islington, District Dispensary, Mr. James Hassall France, St. George's, Hanover Square, District Dispensary, and Mr. Herbert Skinner, Ph.C., Great Northern Central Hospital. Mr. Lindsey and Mr. France, as chairman and secretary respectively of the Public Pharmacists' and Dispensers' Association, have been responsible for the successful work of the Association, to which we have previously referred. Mr. W. J. U. Woolcock, barrister-at-law, who is secretary, is an ex-pharmacist of University College Hospital. The Ladies' Committee includes Miss Elsie Wardle, M.P.S., Queen's Hospital for Children, and Miss M. Bedell, Ph.C., Seamen's Hospital, Greenwich. Altogether a creditable list of public-spirited institution workers.

### A THREEPENNY DOCTOR FINED FOR ASSAULT.

An extraordinary case of assault led to the conviction of a medical practitioner at North London Police Court this week. A summons was taken out by two women, one of whom gave evidence to the effect that she went to Mr. H. P. Jelly's surgery on November 16 to have a tooth extracted. Subsequently she discovered that part of the decayed tooth had been left in the jaw and a sound tooth removed. The jaw, she said, was sufficiently injured in addition to necessitate her consulting another medical man. Two days later she visited the surgery again with her sister, arriving there at 9.30 in the evening, to get an explanation. The defendant retorted that he knew perfectly well what he had done, and that he did not care for her or for five hundred medical men. Then he locked the door of the consulting room and shouted, "Clear out or I will throw you out." He then opened a door into a dark passage and told her to go out that way. She refused, whereupon Mr. Jelly called in a constable and said, "Fling these women out." As the constable would not do so, the doctor struck her sister in the mouth. The witness and the constable ran to her assistance, but the doctor threw the witness down, and the constable had to push him aside before she could get up. The defendant stated that he told the women to leave at once because they had come into his consulting room without paying and in front of eighty patients. Both the complainants struck him, but he was too busy a man to trouble about summoning them. The magistrate sentenced the defendant to pay a fine of £1 in each case, with costs—amounting to £6 4s. in all—or to undergo 14 days' imprisonment. The defendant's qualification appears to be the L.M.S.S.A. only. In a case of this kind it will probably strike medical men as forcibly as laymen that the fine was a very light one. There is no need to repeat how much more severely English law punishes offences against property than offences against life.

### THE RIGHTS OF STATE-INSURED WORKMEN.

A correspondence is being carried on in the *Leeds Mercury* which illustrates so well the confusion existing in the minds of the public, and even of certain institutional workers, on the relation of State-insured persons to the voluntary hospitals, that it is well worth while to deal with the principal points here. Many people still ask, "Why cannot the voluntary hospitals treat State-insured persons in the same way as they now do friendly society members?" The answer is that the voluntary hospitals exist to assist those self-supporting citizens whose incomes, whether reinforced by prudent assurance or not, are insufficient to keep them in times of illness. Such were many of the friendly society members. The hospitals have never received from them anything like the full cost of their treatment, and their sick pay has never carried with it the *right* of institutional care. With the State-insured person the case is different. Employer and employed have been compelled to provide between them 7d. a week on the sole consideration that the State will furnish medical and sickness benefits to the latter. If the State's solemn undertaking means anything at all, unless National Insurance is to prove an idle pretence, the insured must have institutional in-patient treatment. The right to this every insured person who has been compelled to pay his share of the cost must enforce. With this need created by the State, and already paid for, can anyone suppose that the insured person will stoop to accept charity? So far from being an object of charity, the State has given him a right to expect hospital treatment; having paid in advance he now demands the medical services he urgently requires. The German workmen needed no coaching to grasp the State's obligation to them in this respect, and secured hospital benefit as a right, it being paid for on a fixed scale by their approved societies. Is it conceivable that an Englishman will show less sense of what is due to him, and less independence than his German fellow-worker? So much, then, for the friendly society argument.

### WHAT THE FRIENDLY SOCIETY PATIENT COSTS.

Now how does this question affect the voluntary hospital on its financial side? One of the correspondents in the above controversy ventured the statement that, as the voluntary hospitals are supported very largely by working-men's subscriptions, it would be a very bad day for any voluntary hospital which took Mr. Lloyd George at his word, and told insured persons that in the latter's own words they "had no right to come to the hospitals." Yet what is the position at the General Infirmary in Leeds, the city from which he writes? It appears that at least 50 per cent. of the in-patients at a voluntary hospital like the infirmary are insured persons. The accounts show that 20 per cent. of its income is provided by workpeople, and nearly 33 per cent. by the middle and wealthier classes. It is therefore an easy matter to see how much the workpeople cost the hospital. We can compare this with the amount of their subscriptions. The latter are given as £5,250, or about one-fifth of the hospital's income; their cost to the hospital is £14,700, that is 188½, or half the total number of beds, multiplied by £78, the average cost of each bed to the institution. If such is the present position of the parties, it is clear that if the workpeople insist on their right to treatment under the Act, it can be given through their approved societies by arrangement with the hospitals on a basis of *pro rata* payments sufficient to cover the cost of every patient admitted to the State wards, plus 10 per cent. for the remuneration of the medical staffs. If the workpeople continue their subscriptions, the cost involved to the approved society may be less than two-thirds of the actual cost of providing such hospital treatment. For at present, as we have seen, the workpeople at the General Infirmary, Leeds, provide only one-third of their cost to the hospital. After January 15, if all parties combine on the above plan, only two-thirds or less will fall on the approved society. Their combination would therefore not merely save the voluntary system but increase the value of the Insurance Act enormously.

### THE HOSPITALS' DILEMMA.

It should therefore be clear why the insured person cannot be admitted, or rather, will surely refuse to enter the wards as a charity patient. His hospital needs must be met on a business basis, *i.e.* by *pro rata* payments. For the dilemma is this. Certain working-class people, as already mentioned, say that working-men's support will be withdrawn on the day an insured workman is refused admission. But it may prove equally true that middle-class and benevolent support will be withdrawn the moment its money is diverted from the objects of charity to a man who has paid for and been State-guaranteed adequate medical and sickness benefits. If the whole system of voluntary hospitals, the Insurance Act, and the treatment of the sick workman is not to start asunder like a loosened arch, to the infinite hurt of the sick workman, they must co-operate on the above lines. The fate of all three is in the balance.

### THE INCREASING SALE OF PROPRIETARY MEDICINES.

In spite of the campaign against secret remedies there are no signs of any decrease in their popularity. On the contrary, their sale appears to be increasing, for according to statistics issued by the Board of Customs and Excise the revenue from medicine stamp duty for the year ended March 31, 1912, was £327,657, which is an increase of over £2,000 on the preceding year. It is possible from these figures to obtain an approximate estimate of the sum spent in Great Britain on proprietary medicines, for all shilling packages of such medicine must bear a three-halfpenny stamp. The revenue mentioned is therefore equivalent to over 52,000,000 shilling packages of a selling value of over two and a-half million sterling. This, it should be added, is not an exact estimate, since a fair proportion of the medicines sold are of higher selling value than one shilling a bottle and subject to different rates of duty, but for all practical purposes the estimate is sufficiently correct. The number of licences issued during the year in question to makers or vendors of so-called patent medicines was 43,131, a slight increase on the preceding year. It is, therefore, obvious that the trade is in a flourishing condition.

### BACTERIOLOGY AND ADVERTISING.

The enterprise of a well-known firm of patent-food manufacturers has led to their breaking new ground for the purpose of advertising themselves. This time it is nothing less than entering into competition with the bacteriologists and offering to undertake diagnostic examinations at cut-throat prices. Apparently only the simpler examinations are as yet to be performed for these small fees, but this new competition must soon be keenly felt by the proprietary laboratories, and the poorly paid but highly qualified workers in our large hospitals will doubtless find a good deal of their hardly earned private work diverted from their doors. Is this move a shrewd anticipation of the demands for more exact diagnosis which the Chancellor of the Exchequer is seeking to thrust on the profession? We should very much like to know the status and qualification of the persons who are doing this given-away-with-a-pound-of-tea sort of bacteriology.

### RETIREMENT OF A FORTY YEAR'S HOSPITAL SECRETARY.

The retirement is announced of Mr. Henry Johnston, who, ever since the opening of the Western Infirmary, Glasgow, in 1874, has been its secretary and treasurer. How much the institution has developed since its beginning may be judged from the fact that it began with 150 beds—there are 600 to-day—and last year the number of in-patients was something like 9,000. In every step of the progress of which these figures mark the first and latest stages Mr. Johnston has played an active part; and what an amount of work these developments have entailed will need no elaboration to hospital officials, each of whom has an example in his own institution's growth. In spite of this vast extension of scope, however, Mr. Johnston has not confined his energies to the work of the Western Infirmary. He has held, in addition, various other posts connected with several of the many sides of hospital work. For instance, he has been the secretary of the Hospital Saturday Fund, and has acted as treasurer and secretary of the Lady Hozier Home at Lanark. Apart from this he has found his relaxation in other forms of work, of which his post as honorary secretary to the choral orchestral scheme in Glasgow is a typical example. In this Mr. Johnston illustrates one of the best sides of hospital life—the sympathy, namely, which it excites among hospital men with outside activities of all kinds. Though one of the busiest lives in the world is that of the hospital secretary, few professions can provide finer examples of many-sidedness among their members. Mr. Henry Johnston, we note, will be succeeded in the posts of secretary and treasurer by his son, Mr. John Matheson Johnston, who, we understand, will step into his father's place at the beginning of December.

### JOINT COLLECTION SCHEMES.

Many attempts have been made to centralise the collection of hospital subscriptions, and the latest convert to this idea appears to be Aberdeen, which has decided to put into force next January the Lord Provost's suggestion on this head. The joint collection scheme is not a pooling arrangement, and its supporters claim that Liverpool and Glasgow have proved its success for over a quarter of a century. Its advantages are given as the abolishing of individual canvassers and the risks of fraud that have been found to attend this form of raising money, and the simultaneous presentment to prospective subscribers of all the charities of the city. Early every year a booklet is issued to all past or possible subscribers, containing a subscription sheet with a space allotted to each charity and a brief notice of each. The central treasurer invites subscriptions, and, according to the instructions of the subscribers, hands his gift to whichever institution is named. The working expenses appear to be covered by a levy varying from 1 to 4 per cent. upon the total collected. The central committee has received the support of many local hospitals, and an executive committee has been formed. Though the scheme does not preclude individual subscriptions from being sent to separate hospitals, two institutions—the Royal Infirmary and the Sick Children's Hospital—have felt unable to endorse the plan. The reasons which they give are worth notice. The Infirmary fears lest the important part of its income, which is derived from church subscriptions, would suffer; while the Sick Children's Hospital collects its subscriptions by voluntary canvassers without any cost to itself. Generally speaking, with all respect to the instances quoted in favour of a joint scheme, the system which brings out the individual character and needs of each hospital has, we are sure, worked out best.

### THE CHELSEA HOSPITAL DINNER.

We are glad to supplement our account last week of the rebuilding of Chelsea Hospital for Women with the announcement that over £8,000 resulted from the dinner in aid of the Rebuilding Fund that was held last week. Viscount Castlereagh, the hospital's president, and the Countess of Ilchester, president of the Ladies' Committee, presided. The former, in proposing the toast of the hospital, referred to the difficulties encountered by charitable effort, and said that in the great combination adumbrated in the Insurance Act of contributor, employer, and the State must lie the essentials for meeting voluntary effort in supplying a solution to the problem. He went on to say that the whole question was at a standstill because the Insurance Act was the bauble of the party system, and for the moment they must concentrate on making the rebuilding scheme a success. This attitude, we believe, is widely shared by individual institutions, which would be only too thankful, if they could, to throw over the position created by the Insurance Act and to go on as before. We have, however, insisted that an interval of six months is not a permanent interval, and that no further time must be lost by hospital managers in making their combined demands heard. The line which these should take we continue to reiterate, and in the meantime we congratulate the Chelsea Hospital on the well-deserved success of their dinner.

### THE PUBLIC AND TROPICAL MEDICINE.

It is interesting to know that £48,000, or approximately half of the desired sum which Mr. Austen Chamberlain set out to raise for the London School of Tropical Medicine, has been subscribed already. Fortunately for the school and for this fund the recent sanification of Panama has brought home to the minds of the most careless of newspaper readers the immense practical results which the application of the lessons learnt in schools of tropical medicine have already achieved. Panama has fortunately proved to the most ignorant mind the importance of this study, and the fund which Mr. Chamberlain is raising has doubtless benefited, and we hope will continue to do so, from this magnificent advertisement. Tropical medicine has now won a place beside those three favourite public subjects for endowment: children's hospitals, cancer research, and tuberculosis prophylaxis. It is fitting to be able to add in this connection that Mr. Austen Chamberlain has been elected a vice-president of the Seamen's Hospital, to which of course the London School of Tropical Medicine is attached.

### THE DOCTORS' CONFERENCES WITH MR. LLOYD GEORGE.

A small committee of the British Medical Association has been discussing the points at issue between the profession and Mr. Lloyd George with the Chancellor during the early part of this week. Mr. Lloyd George was accompanied by Mr. Masterman, and the doctors' representatives were Mr. Verrall, the chairman of representative meetings, Mr. E. B. Turner, deputy-chairman, Dr. J. A. Macdonald, chairman of the council, Dr. R. M. Beaton, London, and Dr. T. Helme, Manchester. After the meeting on Monday the committee conferred with officials of the Insurance Commission, and Dr. Addison, M.P., also joined in the discussions. On Tuesday a further meeting took place, and several concessions have been anticipated when the committee reports the result of the conferences to the State Sickness Insurance Committee of the British Medical Association, which appears in the Association's journal shortly after we have gone to press this week. The committee, it will be remembered, has no power to negotiate with the Government, and any concessions obtained will be submitted to an individual vote of the members of the profession at divisional meetings. It is, perhaps, worth while to summarise the more sober of the best informed forecasts that have been made as to the concessions which, it is generally felt, have been granted as the result of these meetings. But at present they are forecasts and nothing more. Thus it is said that the duties which the practitioner will not be required to render under the Act will be more clearly defined; that the Government's suggested committee of complaints will be abandoned or much modified to meet the profession's wishes; that some concession on the mileage question will be granted; that in rural districts where no chemist is at hand the doctor may be allowed to dispense; that on the £2 income limit there will not be any concession; and that all thought of a State Medical Service will be abandoned. By the time that this issue is in the hands of our readers they will be able to test these prophesies for themselves.

### SIR DONALD MACALISTER AND INSTITUTIONAL TREATMENT.

In delivering his presidential address at the ninety-sixth session of the General Medical Council on Tuesday, Sir Donald MacAlister said that the committee deputed to consider the Insurance Act had reiterated the Council's opinion that in the absence of any sufficient provision for the "institutional" treatment of insured persons, the existing facilities for the study of clinical medicine, surgery, and midwifery might be seriously endangered. If this were so, he went on, the efficiency of medical education, and consequently of medical practice among all sections of the public, might be impaired. The Commissioners had intimated that the committee's suggestions were found useful, and that, apart from the question of hospital facilities, which the Commissioners appeared to regard as outside the present Act, effect was given to these suggestions in the provisional regulations. He suggested the expediency of widening the terms of reference to the committee, so that it might act and report on any matter arising out of the Act which touched the Council's functions. The ominous vagueness of the Commissioners on " the question of hospital facilities " is enough in itself to show that the hospitals must combine to formulate their own scheme, if a way is to be found out of the present confusion.

### THE VALUE OF FIRE INQUESTS.

The opening of an inquiry by the Home Office into the precautions necessary in the use of celluloid, and the Committee's request for evidence and information, aptly illustrate the value of fire inquests in drawing attention to special dangers of this kind. In addressing the members of the Insurance Institute of London last week on the working and results of the City of London Fire Inquests Act, Dr. Waldo, the City Coroner, said that the facts favoured an extension of the principle of fire inquests to the whole country, and that such an extension would restore to the office of Coroner one of his most valuable public functions. The London County Council, he proceeded, were averse to this proposal, although it had been expressly made by Lord Gladstone's Departmental Committee, which reported in 1909. Surely neither the London County Council, nor for the matter of that any local authority, nor special department as suggested by the County Council, he urged, ought to direct in what particular case fire inquests were or were not to be held, since either of these might possibly be an interested party. The above contentions were illustrated by some interesting figures, of which we have space to quote only the most striking—namely, that in 1911, out of 145 fires reported, in only 22 cases did the cause remain undiscovered. We agree with the speaker that the power to hold fire inquests acts, and has been proved to act, as a deterrent against arson and fraud. Neither of these specific dangers, fortunately, threatens the hospital of to-day in the same sense as other buildings. Still, the fires that do occur in hospitals from time to time generally give proofs that for some reason or other people, even in responsible positions, are more indifferent to the chance of fire than to almost any other risk. If this were not so, why even in hospitals are fires due to such avoidable causes as a lack of water?—to quote a recent case. We are therefore always glad to impress hospital officials with every side of the important question of fire prevention. The fire inquest is certainly one.

### THE INTERNATIONAL CONGRESS OF MEDICINE.

To look ahead for nine months seems a long time, however short the same period may be to look back upon, but we make no apology for reminding our readers that next August the International Congress of Medicine will be held. It will be also one of the largest international gatherings of recent years. Five thousand medical men are expected to be present as delegates. There are twenty-six sections, most of which will meet daily in various parts of London, but the mass meetings, as the numbers indeed necessitate, will be held in the Albert Hall. The Congress assembles once in every four years, and was last held in Budapest in 1909. The Congress of 1881, over which Sir James Paget presided, was notable for the presence of Huxley, who spoke on the subject of heredity, and of Pasteur. The Congress will be presided over by Sir Thomas Barlow. The general addresses to the full Congress will be given by Professor Chauffard, of Paris, on medicine; Geheimrat Professor Paul Ehrlich, of Frankfurt, on pathology; Mr. John Burns, M.P., on public health; Professor Harvey Cushing, of Harvard University, on surgery; and Mr. W. Bateson, F.R.S., on heredity. A fund is being raised to meet the expenses of the meeting, which are expected to amount to £8,000.

### FINANCE AND THE DERBY INFIRMARY.

The hundred-and-third anniversary of the Derbyshire Royal Infirmary was held on the same day as its annual meeting. Speaking at the latter Mr. G. Herbert Strutt, who presided, said that the Insurance Act had already cost the hospital £28, and would probably cost it about £120 a year. There has been an actual deficit of £284 on the year's work, but it must be remembered that the board estimates the cost of the coal strike to the hospital as £150. It is from the Royal Infirmary, it will be recalled, that Mr. Welsh, the hospital's house surgeon, recently obtained leave to volunteer for service in the Balkan States. Financial matters, however, were in great prominence. The building debt of £1,120 which has been hanging over the institution for a long time has been paid off by calling in a mortgage on a property in Derby. In spite of the difficult outlook, Mr. Strutt expressed the belief that the hospital might hope for a better financial future.

### SURPRISE ROYAL VISIT TO AN ACCIDENT HOSPITAL.

In spite of the wet and blusterous weather which attended the opening days of their Majesties' visit to the Duke and Duchess of Portland at Welbeck Abbey, the Queen, accompanied by the Duchess and the Marquis de Soveral, paid a surprise visit to the Victoria Hospital, Welbeck, very soon after her arrival. Among the patients were two quarrymen, who had been injured in an explosion. Indeed, the small institution, which has only fourteen beds, is largely designed to serve for local accidents of this kind, and that her Majesty has honoured it with a special visit is the best proof of the regard in which she holds every institution under the voluntary system, whether large or small.

### THE LAMBETH DISPENSARY AND ITS CRITICS.

The Lambeth Borough Council are making their arrangements for a tuberculosis dispensary, which is to be situated in Effra Road, Brixton. The position is criticised as a most unfortunate one. The bulk of the cases of tuberculosis come from the part of the borough situated close to the riverside, and, as they are amongst the poorer inhabitants, it will be a source of trouble to them to have to journey to the dispensary some half-hour's tramway ride away. The Council has, moreover, fixed the commencing salary of the tuberculosis officer at £300 a year, but the Local Government Board has written doubting whether the proposed commencing salary of £300 per annum would be sufficient to attract medical men possessing the necessary qualifications and experience. Dr. McKeith, M.D., C.M., who was recently returned as a member of the Council, supported the views of the Local Government Board. The Council, however, adhered to its resolution. So we fear it must be said that the project of the Lambeth Borough Council has been criticised adversely all round.

### HOSPITALS AND DOLLS.

On Wednesday, December 18, as every hospital worker ought to remember with genuine gratitude, the *Truth* Doll and Toy Show opens at the Albert Hall. For thirty-two years the *Truth* Toy Fund has provided dolls and toys at Christmas for the children in the workhouses and Poor-Law infirmaries and many hospitals. That is, for the most fun-forgotten children in the country. It was the first newspaper to start the idea; it has done thereby incalculable service to Christmas in the children's wards for over a quarter of a century, and the result is that after having borne the burden and heat of the day, and having popularised the idea of the Toy Fund, and, in fact, shown how it could be organised successfully, another newspaper is stepping in to endeavour by a rival exhibition to divide the honours with the *Truth* Toy Show. We hope and believe that there is enough gratitude in the hospital world to make this attempt abortive. And we earnestly ask some one person in every institution into which this reminder is brought to make it his or her business to see that nothing is sent to any other toy fund or doll show until the *Truth* Fund has had the first share. The circumstance of this thirty-second exhibition offers a splendid opportunity to every secretary and matron to show in the most practical possible way their gratitude to the premier Toy Show, and to the newspaper which invented the idea by which children for over a generation have received a Christmas gift from *Truth*. It should not be too much to ask those who often have thankfully opened the *Truth* parcels, this year to send a parcel or its equivalent themselves. The time is short, but there is still time to post subscriptions to the Toy Fund or actual dolls to the Editor of *Truth*, Carteret Street, London, S.W.

### INFECTIOUS CASES IN WALSALL HOSPITAL.

The question of dealing with cases of infectious disease occurring within the borough of Walsall, more especially when complicated with conditions requiring surgical interference, is one which for many years has taxed the resources of the local hospital authorities. The borough possesses no hospital for treating infectious disease, except a smallpox hospital and an old epidemic hospital, used chiefly for treating cases of typhoid fever. The absence of a fever hospital in Walsall has caused a great deal of anxiety. At the hospital it has not always been possible to place an isolation ward at the disposal of such cases as have developed scarlet fever or diphtheria after admission, and, moreover, an isolation ward in a general hospital is not intended for this purpose; consequently, with one or two exceptions, when through the courtesy of the town clerk and the local health authority patients have been received into the typhoid hospital, these cases have been sent to their own, more often than not, quite unsuitable homes. The hospital has also been caused considerable inconvenience through being called upon to nurse cases of diphtheria which have been brought in for the performance of tracheotomy. At length an arrangement has been entered into between the local authority and the committee of the Walsall and District Hospital whereby cases of diphtheria likely to require surgical interference will be received into the hospital and placed in one of the detached wards which was closed through lack of funds in 1907, and which was formally placed at the disposal of the health committee on October 1 last. By way of recognition, the latter have agreed to pay to the hospital an annual subscription of £80 and £3 3s. per week per case. Though the medical officer of health will have the privilege of visiting this ward, the full responsibility for the care of the cases will devolve upon the board of the hospital in conjunction with their honorary medical staff. It is reported that arrangements are also being made by the health authority for dealing with cases of scarlet fever requiring surgical attention at the epidemic hospital referred to.

### THIS WEEK'S DRUG MARKET.

With the exception of the usual demand for drugs in request at this season of the year, business continues dull. There is still practically no change in the position of opium and its derivatives. Cod-liver oil barely maintains its price from week to week. Oil of cloves is dearer. The values of ergot of rye, glycerine, eucalyptus oil, and olive oil are well maintained. Unfavourable reports of the olive crops have been received from many districts, and the probability is that prices will become higher. There are still offers of oil of male fern of very doubtful quality, and buyers of this drug should be satisfied that they are obtaining the genuine article. There is as yet no change in the price of potassium bromide and other salts of bromine, but an advance is not unlikely.

### TO OUR READERS.

Contributions are specially invited from any of our readers to these columns. They should deal with topical subjects and news. They must be authenticated for the information of the Editor only. The minimum payment if published is 5s. There is no hard-and-fast rule as to space, but notes of about twenty lines in length are preferred.

